# A Systematic Review and Meta-Analysis of the Prevalence of Methamphetamine Abuse in Iranian High School Students

**DOI:** 10.25122/jml-2018-0020

**Published:** 2018

**Authors:** Mehdi Sayyah, Kiarash Shirbandi, Fatemeh Javanmardi, Fakher Rahim

**Affiliations:** 1. Education Development Center, Ahvaz Jundishapur University of Medical Sciences, Ahvaz, Iran; 2. Systematic Review and Meta-analysis Expert Group (SRMEG), Universal Scientific Education and Research Network (USERN), Tehran, Iran; 3. Department of Biostatistics, Health Sciences School, Ahvaz Jundishapur University of Medical Sciences, Ahvaz, Iran; 4. Health Research Institute, Thalassemia and Hemoglobinopathies Research Center, Ahvaz Jundishapur University of Medical Sciences, Ahvaz, Iran

**Keywords:** Drugs, Methamphetamine, Meta-analysis, Iran

## Abstract

**Background and Aim:** Abuse of drugs such as methamphetamine is one of the most important problems in high-school children and adolescents according to the World Health Organization (WHO), which has mentioned it as a concerning event in the world. Therefore, the purpose of this study was to estimate the prevalence of methamphetamine abuse in Iranian students using the meta-analysis method on studies conducted in Iran.

**Materials and Methods:** To select the studies, a systematic search was performed on leading databases, including ISI web of science, PubMed, Scopus, Embase, PsycINFO and PROSPERO with no language limits from their inception to 31 Jan 2018. Furthermore, local databases, including SID, Magiran, and IRANDOC were searched systematically using both Persian and English languages from their inception to 31 Jan 2018.

**Results:** We found a total of 828 potentially relevant studies, of which 30 met our criteria, and 7 articles (7452 students, 3063 females and 4389 males) were included. The pooled prevalence of methamphetamine use in Iranian students was 0.016% (95% CI: 0.06-0.041, P=0.00). The heterogeneity was low (I^2^ =44.41, d.f = 6), which shows that about 94.41 of the total observed variance was true variance between the studies.

**Conclusion:** Although the prevalence of methamphetamine in high-school students is lower than in other drugs, a growing pattern in Iranian high-school students in recent years is a serious warning to authorities and families. Increasing families and students’ knowledge about the harmful effects of this drug can be an effective approach to reducing its prevalence in young people, especially high-school students.

## Introduction

Substance abuse is one of the most important behavioral problems in today’s community and society. The World Health Organization (WHO) has mentioned this problem as one of the most important issues facing societies in the world [1]. Undoubtedly, some teenagers and adolescents, without sufficient knowledge, use drugs to stop feeling sad. Unfortunately, the census and statistical reports in the country indicate the depth of the disaster [2]. Out of high-risk age groups, adolescents and school-children for particular reasons, including excitement of self-centered emotions, the sense of independence, and the tensions of growth, are more prone to drug abuse problems such as delinquency, drug use in peer groups, educational stagnation, suicide and continued use in adulthood [3].

Drug and substance abuse, such as use of methamphetamine, can be described as any consumption of these drugs or substances to change mood and behavior. In spite of various educational, health, and judicial efforts in the fight against drug abuse, it remains a considerable health and social problem [4]. Epidemiological evidence suggests that adolescents, especially boys, are more vulnerable to drug addiction than adults [5]. Various factors such as age, gender, race and nationality, religious beliefs, material availability, community attitudes toward substance abuse, geographical location, peer pressure and the presence of an addicted person in the adolescent family are the reasons for more likely developing substance use disorders, including methamphetamine, in this age group [4, 6].

According to the 2003 United Nations Office on Drugs and Crime (UNODC), the abuse of methamphetamine is increasing [7, 8]. The report shows that more than 35 million people use methamphetamine, while the number of users of cocaine and heroin is estimated to be 15 and 10 million, respectively [9]. Iran has long been facing substance abuse, but today transition from traditional to synthetic drugs has become a new problem [10]. This phenomenon is a new challenge because these drugs, unlike traditional ones, do not enter the country at a specific geographical boundary, but are produced in small, unsafe local laboratories [11].

In the last decades, drug abuse and dependence on synthetic substances, especially methamphetamine, have become serious problems and a health emergency in our country, which has led many authorities at all levels to actionable decision-making [12, 13]. The pieces of evidence show that out of last year’s high-school students, methamphetamine abusers ranged from 5% to 12%, and one out of every 10 students experienced it. Most methamphetamine users in the world were in the age group of 10 to 18 years old [14, 15]. Methamphetamine abuse causes unpleasant effects such as hyperthermia, dry mouth, tachycardia, nausea, anxiety, sleep disturbances, depression, and may even result in death [16]. The risk of death after the first abuse of methamphetamine ranges from one case in every 2,000 to one in every 50,000 consumers [17].

To show the methamphetamine abuse variations both over time and space in Iranian high school children, in this systematic review and meta-analysis we assessed the latest epidemiological evidence on methamphetamine abuse and briefly discussed the subpopulations that are at high risk of impairment due to abuse of this drug. Therefore, considering the importance of the discussed issues, this study aimed to estimate the prevalence of methamphetamine abuse in Iranian students using the meta-analysis method on studies conducted in Iran.

## Materials and Methods

### Study Design

This meta-analysis was performed in accordance with the Meta-analysis of Observational Studies in Epidemiology (MOOSE) [18] and PRISMA guidelines [19].

### Sources of Information

To select the studies, a systematic search was performed on leading databases, including ISI web of science, PubMed, Scopus, Embase, PsycINFO and PROSPERO with no language limits from their inception to 31 Jan 2018. Furthermore, local databases, including SID (Scientific Information database, http://sid.ir/), Magiran (http://www.magiran.com/), and IRANDOC (http://irandoc.ac.ir/) were searched systematically using both Persian and English languages from their inception to 31 Jan 2018. The search was performed using the following keywords: “methamphetamine”, “high school students”, and “epidemiology or prevalence or incidence”. In addition, to find more eligible studies, the reference lists of relevant publications were manually searched.

### Study Selection

Studies that fulfilled defined criteria, including observational studies (prospective cohort, retrospective cohort, case-control, or cross-sectional) and reporting the prevalence of methamphetamine abuse among high school children, of which the full-text was accessible, were considered in the meta-analysis. Review articles, technical reports, working papers, conference proceedings, and other ‘grey’ literature, were excluded.

### Data Extraction

Data were collected by a data extraction form, including first author name, publication year, location, study design, sample size, demographic characteristics such as age and sex, and criteria for enrolling. Two authors (F.R. and K.Sh.) separately extracted the information of interest from studies. We contacted the authors of the eligible articles for missing data, if necessary.

### Quality Assessment

Quality assessment is a structured list of traits or items that are extracted or determinable from a published paper in order to evaluate the accuracy of study results and the data reported in the study. In this article, the quality of selected studies was assessed using a ten-item Joanna Briggs Institute critical appraisal tool [20, 21]. In short, this tool assesses the prevalence study criteria, including representativeness, sample size, recruitment, setting, condition measured reliably and objectively, data coverage of the identified sample, description and reporting of study subjects, statistical analysis, and confounding factor.

### Publication Bias

Publication bias must be taken particularly seriously, as it presents perhaps the greatest threat to the validity of meta-analysis. Several reasons may affect publication bias such as non-significant treatment effects, more citation of some studies or inaccessible languages. Various methods have been developed to detect publication bias in the meta-analysis, such as graphical approaches and formal statistical tests, but Begg-Mazumdar and Egger’s regression methods give better results. In fact, this specific test was used to demonstrate that there is no evidence that the results were significantly affected by publication bias. Asymmetry in a funnel plot of study size against treatment effect is often used to identify such bias [22]. Here, to observe the possibility for *publication bias*, visual inspection of funnel plots and the Egger test were used [23].

### Sensitivity Analysis

We conducted a series of sensitivity analyses to evaluate the robustness of our results, such as the influence analysis that involves eliminating a single study from the meta-analysis at a time to judge whether a study mainly contributes to the overall prevalence estimate [24, 25].

### Statistical Analysis

We pooled the prevalence estimates of each study using a random-effects model for meta-analysis [26]. Heterogeneity was tested using both *I*^2^ statistic and Chi-square test. *I*^2^>50% or *P*<0.05 were considered to exhibit significant heterogeneity. We tried to clarify heterogeneity using an unrestricted maximum likelihood mixed effects meta-regression analysis [27]. Review manager 5.3 (Nordic Cochrane Centre, The Cochrane Collaboration, Copenhagen) was used to provide pooled estimations, with corresponding 95% CI and plots. To determine the heterogeneity between the studies, Q, T^2^, and I^2^ indices were used. The Q index determines the statistical significance for heterogeneity and T^2^ and I^2^ indices estimate the effect of inhomogeneity. Also, the inverse method was used to estimate the variance and weight for each study (a split on the intra-group variance of each study + variance between groups of studies). Stata version 11.0 and Comprehensive Meta-Analysis Version 3 software were used in the meta-analysis. A P-value less than 0.05 was considered statistically significant.

## Results

**Figure 1:**
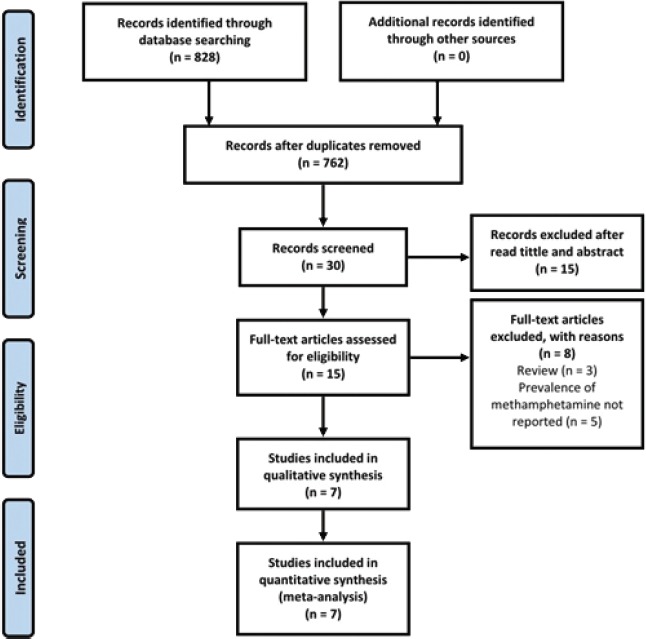
Study Flow Diagram showing how to extract articles about the incidence of methamphetamine in Iranian male students

We found a total of 828 potentially relevant studies, of which 30 met our criteria and 7 articles were included (7452 students, 3063 females and 4389 males) [28–34] (Figure 1). The subjects’ mean age was 16.38 ± 1.02 years.

Of the 828 published in this study, the highest prevalence of methamphetamine was 12.7% in Yasuj, an industrial city in southwestern Iran; while the lowest prevalence was 0.4% in the Karaj province, located in northern Iran (Table 1).

**Table 1:** Characteristic of included studies

Author	Year of study	Type of study	Sample size	Age mean (range)	Female N (%)	Prevalence in boy N (%)	Prevalence in girl N (%)	Overall prevalence N (%)	Study location
**Ahmadi and Hasani 2003[28]**	2000	Cross sectional	397	16.56 ± 1.29 (13–24)	200 (50.37)	3 (1.5)	0	3 (0.8)	Shiraz
**Mohammadkhani et al., 2011[32]**	2007	Cross sectional	2538	16.02 ± 0.98 (13-18)	1255 (49.4)	5 (0.4)	3 (0.2)	8 (0.3)	Iran
**Alaei et al., 2011[29]**	2011	Cross sectional	447	16.5 ±1.29 (13–24)	239 (53.46)	2 (1.1)	0	2 (0.4)	Karaj
**Baheiraei et al., 2013[30]**	2010	Cross sectional	1201	16.74 ± 1.09 (15–18)	609 (50.70)	19 (3.30)	13 (2.20)	32 (2.70)	Tehran
**Bidel et al., 2014[31]**	2011	Cross sectional	937	16.2 ± 0.5 (12–22)	–	20 (2.1)	–	20 (2.1)	Ilam
**Nazarzadeh et al., 2014[33]**	2013	Cross sectional	1524	16.02 ± 0.98 (14–18)	760 (49.86)	–	–	32 (2.10)	Ilam
**Ataee et al., 2014[34]**	2013	Cross sectional	408	16.62 ± 0.89 (13–19)	–	52 (12.7)	–	52 (12.7)	Yasuj

**Figure 2:**
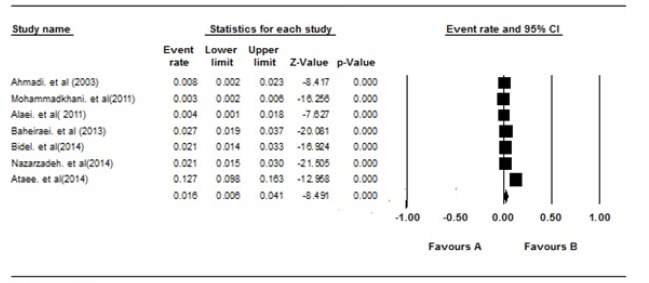
The prevalence of methamphetamine use in Iranian students over the years 2000-2018

The pooled prevalence of methamphetamine use in Iranian students was 0.016% (95% CI: 0.06-0.041, P=0.00). The heterogeneity was high (I^2^ =44.41, d.f = 6), which shows that about 94.41% of the total observed variance was true variance between the studies (Figure 2).

Considering included studies, 4 studies reported a prevalence of methamphetamine use in Iranian male students [28, 29, 31, 34], and 2 studies reported a prevalence of methamphetamine use in both Iranian male and female students [30, 32]. According to subgroup analysis, the prevalence of methamphetamine use in Iranian male students did not differ significantly from overall analysis, but in Iranian female students it was 0.007% (95% CI: 0.04-0.012, P=0.00) (Table 2).

**Table 2:** Summary of overall and sub-group meta-analysis

Prevalence	No. of studies	Prevalence of mutation(95%CI)	*I^2^%*	Heterogeneity test		Egger test	
				Z	P	t	P
Overall prevalence	7	0.016 (0.06–0.041)	44.41	107.39	0.00	–1.16	0.298
Prevalence of Boys	6	0.013 (0.004–0.046)	23.20	86.16	0.00	–1.07	0.346
Prevalence of Girls	2	0.007 (0.00–0.00)	15.01	19.062	0.00	–2.23	0.416

**Figure 3:**
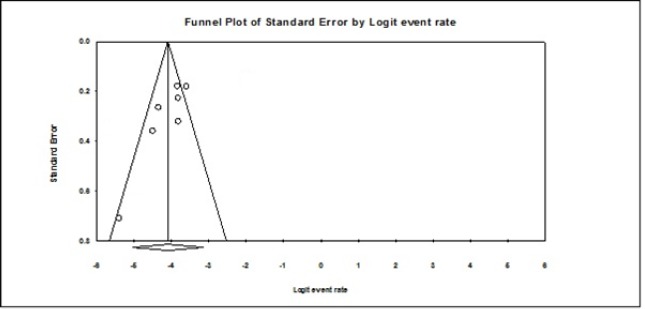
Funnel plot assessing risk of publication bias in the meta-analysis.

In the sensitivity analysis, when each study was removed in turn, the pooled prevalence of the rest of the studies did not alter significantly. No evidence of publication bias was found. (Egger’s test: *P*=0.285 and Begg’s test: *P*=0.837) (Figure 3).

## Discussion

Students with wrong beliefs typically use methamphetamine to remain awake so that they can study all night for exams or have the energy to work a part or full-time job at the same time as attending school and focusing on their studies. The main objective of this study was to determine the prevalence of methamphetamine use in Iranian students using a meta-analysis study in Iran. According to the results, the prevalence of methamphetamine use in Iranian students was 0.02%, which shows that the prevalence of methamphetamine use in Iranian high-school students was considerably low in our study. Longitudinal studies on the prevalence of substance abuse in Iranian high-school students also reported considerably low prevalence among boys and girls [35–37]. Comparing to other countries, the prevalence of substance abuse, especially methamphetamine, was significantly lower in Iranian high-school students [38–40]. According to a published study on the prevalence of methamphetamine and its derivatives in North America, it was 0.8% in Canada, 0.1% in Mexico, and 1.4% in the United States [41], which was slightly higher than the current meta-analysis. The differences in observation in various countries and cities in terms of the prevalence of methamphetamine can be owed to the measurement tools used in studies, the changing pattern of drug use over different years, the awareness of the use of substances among students, the different cultural and social environments, the lack of a proper reporting system, may justify the heterogeneity between the estimated outbreak of studies [42]. In the study on methamphetamine consumption in the city of Nagoya, Japan, the prevalence was of 6.8%, and it was about 4 times higher in boys than in girls [43]. The present meta-analysis also showed a higher prevalence of methamphetamine abuse in boys than girls, but the findings were not significant due to small published studies.

### Limitations

The given findings of the present meta-analysis, as expected in all school-based surveys, are subject to the limitations associated with self-reported studies, and are expected to underreport stigmatized methamphetamine abuse. Our findings may be difficult to generalize to adolescence taking into consideration an inconsistent connection to the school. Besides, the prevalence of methamphetamine abuse among high-school students may vary geographically. Another critical limitation is that an assessment of the rates of substance abuse in various countries is important and many areas underestimate the level of methamphetamine abuse. Therefore, it would be much better if a better understanding of the reasons for methamphetamine use could be reached. Usually, a survey needs to understand better why methamphetamine-dependents continue to use methamphetamine. Thus, because in the selected pieces of evidence there was no such data available, it provided a limitation to our study.

## Conclusion

Although the prevalence of methamphetamine in high-school students is lower than that of other drugs, an increasing pattern in Iranian high-school students in recent years is a serious warning to authorities and families. Increasing families’ and students’ knowledge about the harmful effects of this drug can be an effective approach to reducing its prevalence in young people, especially high-school students. Given the health-related risk factors of substance abuse among students and adolescents, including age and peer pressure, the students, teachers, parents and health authorities are more aware of the effects of drug abuse. In this regard, management measures, social support programs, school environment improvement, and training in coping with drug problems, controlling and preventing synthetic drugs should be considered. The risk factors for the use of methamphetamine in the form of cohort studies are suggested for further research. Education against substance abuse is only effective when those educating understand that students are probably not using methamphetamine just because they are “sad”. Students typically use methamphetamine to remain awake so that they can study all night for exams or so that they can both study and go out with friends. Students use methamphetamine to have the energy to work a part or full-time job at the same time as attending school. Also, students with ADD self-medicate with methamphetamine to help them focus on their studies. Methamphetamine can also be used to control body weight.

## Conflict of Interest

The authors confirm that there are no conflicts of interest.
